# A spurious elevation of serum creatinine level in a patient with Crohn’s disease without histologic kidney damage: a case report and review of the literature

**DOI:** 10.1590/2175-8239-JBN-2023-0071en

**Published:** 2023-11-03

**Authors:** Sul A Lee, Gearoid Michael McMahon

**Affiliations:** 1 Brigham and Women’s Hospital, Division of Nephrology, Boston, MA, USA.; 2 Harvard Medical School, Boston, MA, USA.

**Keywords:** Inflammatory Bowel Diseases, Crohn Disease, Serum Creatinine, Diet, High-Protein, Doenças Inflamatórias Intestinais, Doença de Crohn, Creatinina Sérica, Dieta Rica em Proteínas

## Abstract

Patients with inflammatory bowel disease (IBD) are prone to develop kidney injury. Renal involvement in IBD patients is usually diagnosed by the measurement of serum creatinine and the estimation of the glomerular filtration rate. We describe a patient with IBD who presented with large fluctuations in his serum creatinine level (~3.0-fold) without significant histologic abnormalities and with a normal cystatin C level. This appears to be related to a high-protein diet and intermittent fasting. Even though the impact of a high-protein diet on mild elevations of the serum creatinine level has been described, large fluctuations in serum creatinine from diet alone, as seen in this case, have never been reported, raising the question about the potential contribution of inflamed bowel on gut absorption or metabolism of creatinine. This case highlights the importance of a detailed history, including the dietary habits, when encountering a patient with increased serum creatinine level, and careful interpretation of serum creatinine in a patient with a creatinine high-protein diet or underlying IBD.

## RESUMO

Pacientes com doença inflamatória intestinal (DII) são propensos a desenvolver lesão renal. O envolvimento renal em pacientes com DII é geralmente diagnosticado pela medição da creatinina sérica e pela estimativa da taxa de filtração glomerular. Descrevemos um paciente com DII que apresentou grandes flutuações em seu nível de creatinina sérica (~3,0 vezes) sem anormalidades histológicas significativas e com nível normal de cistatina C. Isso parece estar relacionado a uma dieta rica em proteínas e jejum intermitente. Ainda que o impacto de uma dieta rica em proteínas em elevações leves do nível de creatinina sérica tenha sido descrito, nunca foram relatadas grandes flutuações na creatinina sérica apenas devido à dieta, como observado neste caso, o que levanta a questão sobre a possível contribuição do intestino inflamado na absorção intestinal ou no metabolismo da creatinina. Esse caso destaca a importância de um histórico detalhado, incluindo os hábitos alimentares, ao se deparar com um paciente com nível de creatinina sérica aumentado, e a interpretação cuidadosa da creatinina sérica em um paciente com dieta rica em proteínas ou DII subjacente.

## Introduction

Renal involvement in patients with inflammatory bowel disease (IBD) is not uncommon^1^. IBD patients can develop dehydration from ongoing diarrhea or blood loss. Parenchymal kidney damage can develop from immune-related glomerular or tubular damage, systemic inflammatory conditions, or side effects from the IBD treatment drugs^2^. IBD patients are at risk of developing nephrolithiasis or retroperitoneal fibrosis, which can cause post-renal kidney damage. Prompt recognition of kidney dysfunction and vigilant differential diagnosis are very important to halt or reverse the ongoing kidney damage.

Renal dysfunction in the IBD population is typically diagnosed by measuring serum creatinine concentration. The use of serum creatinine level is the most common means of assessing kidney function^3^. However, serum creatinine level can be influenced by multiple factors unrelated to glomerular filtration rate (GFR) including muscle mass, frailty, drug use, and diet^4^. Herein, we describe a case of recurrent large variations in serum creatinine concentration without histologic evidence of kidney injury in a patient with Crohn’s disease.

## Case Presentation

A 29-year-old male presented to the nephrology outpatient clinic for assessment of abnormal kidney function. He had a history of Crohn’s disease, diagnosed 5 years earlier, and hyperlipidemia. His baseline kidney function was normal prior to this presentation (0.6–0.9 mg/dL) apart from a single episode of acute kidney injury (peak creatinine 1.92 mg/dL) 2 years ago, which was attributed to dehydration from diarrhea.

**Figure 1.**
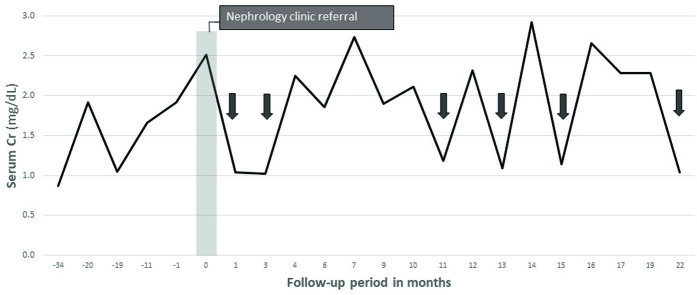
Trends of serum creatinine levels (solid line) and blood urea nitrogen levels (dashed line) during the follow-up period. Results obtained after at least 8 hours of fasting are marked with arrows.

He was found to have an elevated serum creatinine of 1.92 mg/dL [estimated GFR (eGFR) 46 mL/min/1.73m^2^] during a routine visit to the gastroenterology clinic, which increased to 2.51 mg/dL (eGFR 33 mL/min/1.73m^2^) on repeat, and he was referred to the nephrology clinic for further evaluation. He had intermittent diarrhea but reported good hydration and normal oral intake. He had back and left hip pain likely due to radiologically proven early inflammatory sacroiliitis, but he denied any recent use of non-steroidal anti-inflammatory drugs. He had no recent exposure to intravenous contrast or proton pump inhibitors. He had previously been on mesalamine and adalimumab treatment for Crohn’s disease but was not on any medical treatment at the time of referral. He denied herbal or supplement use. He denied any family history of kidney disease or early onset of heart disease. At the initial visit, he was 182.9 cm tall and weighed 64.8 kg (body mass index 19.4 kg/m^2^). His blood pressure was 140/77 mmHg. The physical exam was normal. Urine analysis was negative for blood and protein. Both urine protein (< 4.0 mg/dL) and urine microalbumin (< 1.2 mg/dL) were below the detectable range. Serologic studies including anti-nuclear antibodies, anti-double stranded DNA, cryoglobulins, anti-glomerular basement membrane Ab, anti-phospholipase A2, and rheumatoid factor were negative. C3 (82 mg/dL) and CH50 (36 U/mL) levels were marginally low but C4 level was normal. Serum protein electrophoresis did not detect M-spike, and free kappa/lambda ratio was normal. He was immune to hepatitis B and had no detectable hepatitis C antibodies. Urinary oxalate level was not elevated. Fecal calprotectin level was elevated at 360.5 mcg/g. Renal ultrasound with arteries and veins duplex showed normal kidney size (right, 13.8 cm; left, 12.0 cm), normal echogenicity, and no Doppler evidence of hemodynamically significant stenosis. There was no evidence of stones or hydronephrosis. Renasight™ comprehensive kidney gene panel was negative for any pathogenic mutations. His serum creatinine level fluctuated in the range of 1.0–2.9 mg/dL during the follow-up period while his blood urea nitrogen level remained stably elevated (Figure 1). In the meantime, the patient was treated with vedolizumab monoclonal antibody because of the sustained elevation of calprotectin level and ongoing mild diarrhea, indicating chronic inflammation in the gastrointestinal tract. The treatment course was complicated by the recurrence of clostridium difficile colitis. Given no clear etiology to explain his abnormal renal function, the patient underwent a renal biopsy, which showed no evidence of active glomerulitis, acute interstitial nephritis, or acute tubular necrosis. No calcium oxalates, urates, or phosphates were seen in the sample. Following a more detailed history taking, the patient reported that his serum creatinine level tended to be lower on fasting blood tests but higher when he was not fasting. He also reported an unusually high protein diet, consuming 3-4 pounds of meat daily. Given the discordant cystatin C-based eGFR (> 90 mL/min/1.73 m^2^) compared to creatinine-based eGFR (39 mL/min/1.73 m^2^) and normal kidney biopsy, it seemed that the elevation of serum creatinine level was related to altered gut absorption of creatinine in the setting of his known Crohn’s’ disease and a high-protein diet. The measured creatinine clearance using 24-hour urine collection was 83.12 mL/min/1.73 m^2^ (sample was somewhat undercollected). The patient is currently getting no specific treatment for the elevated creatinine level and remains on a high-protein diet.

## Discussion

Extraintestinal manifestations are common in inflammatory bowel disease patients, most commonly involving the joints, skin, and eyes^5^. Renal involvement in IBD patients can commonly manifest as nephrolithiasis, glomerulonephritis, tubulointerstitial nephritis, and amyloidosis^5,6^. Drugs that are commonly used in IBD patients, including 5-aminosalicylate, cyclosporin A, and tumor necrosis factor alpha inhibitors, are also associated with tubulointerstitial damage. Our patient had a history of Crohn’s disease with extraintestinal manifestations (inflammatory sacroiliitis), ongoing diarrhea, and was on biological agents, putting him at higher risk of developing IBD-related renal insufficiency. However, this patient did not show evidence of pathologic abnormalities on any of the imaging, biopsy, or urinary studies, except for transient microalbuminuria that lasted less than 3 months. Serum cystatin C and 24-hour urine creatinine clearance results were discordant with the elevated serum creatinine level, suggesting that the elevation in serum creatinine had a cause other than his renal function. However, unlike many patients with spurious elevations in creatinine, he did not have an abnormally large muscle mass and was even borderline underweight.

It has long been discussed that a high-protein diet or ingestion of cooked animal meat can elevate serum creatinine levels^7,8,9^. The serum creatinine level changes reported in these studies ranged from 0.1~0.8 mg/dL, or 10–50% above baseline, depending on the amount of ingested protein, which can lead to a misclassification of serum creatinine elevation and falsely lower eGFR. However, our patient showed unusually high serum creatinine level elevation (2.5~3.0 fold) following high protein intake.

Creatine in skeletal muscle of red meat is converted to creatinine during cooking^10,11^. Unlike our old belief that serum creatinine is at steady state with the constant production from skeletal muscle and stable excretion rates through kidney in general population, ingested creatinine from cooked meat is absorbed through the gastrointestinal tract, inducing a transient increase in serum and urinary creatinine levels, as seen in this case. The total amount of creatinine absorption through the gut is determined by the amount of cooked animal meat ingested and the absorptive capacity of the gastrointestinal tract. A previous animal study showed that ingested creatinine is passively absorbed by gastrointestinal absorptive cell junctions^12^. To our knowledge, there has been no specific study on increased gut absorption of creatinine in IBD patients. However, increased tight junction permeability from mucosal immune activation and cytokine release is a well-established concept in IBD patients, the so called ‘leaky gut’^13^, which might have contributed to this patient’s false serum creatinine elevation. Additionally, the gut microbiome can contribute to extrarenal creatinine clearance through creatininase-containing bacteria^14,15^. Altered gut microflora in IBD could have limited creatinine clearance by gut bacteria even though the contribution of gut clearance is usually minimal in patients with preserved renal function. This case report suggests a careful interpretation of creatinine-based eGFR in IBD patients, especially given the increasing popularity of a high-protein diet in IBD patient population, based on several recent studies regarding the potential role of a high-protein intake in alleviating gut inflammation and barrier regulation in IBD patients even though the data are limited^1,16^.

Fasting is not routinely recommended for kidney function tests. This case suggests that physicians should advise their patients to avoid ingestion of cooked meat shortly before serum creatinine measurement and during urine collection of 24-hour urine sample. More careful interpretation of serum creatinine-based eGFR is recommended in IBD patients as their gut creatinine absorption capacity might be increased compared with the general population. A detailed history of the patient’s dietary habits should be obtained during the initial evaluation, given the rise in popularity of high protein diet in the general population. Confirmation of creatinine-based eGFR with other analytical methods, including serum cystatin C or creatinine clearance from 24 hr urine collection, should be considered if patients have an isolated elevation in serum creatinine level without any evidence of pathologic renal damage in urine or imaging studies. Of note, a previous study has shown that serum cystatin C is significantly associated with inflammation independent of kidney function^17^, even though it remains unclear whether the use of cystatin C can lead to an underestimation of renal function in patients with high inflammatory conditions like IBD.

In conclusion, this case demonstrates the importance of a more detailed dietary history and careful interpretation of serum creatinine levels in patients with altered gut mucosal integrity as well as in the general population.

## ABBREVIATIONS

IBD: Inflammatory bowel disease
GFR: Glomerular filtration rate
eGFR: Estimated glomerular filtration rate
